# Supravalvular aortic stenosis in a case of Williams syndrome

**DOI:** 10.11604/pamj.2022.42.170.35211

**Published:** 2022-07-01

**Authors:** Yash Gupte, Sourya Acharya

**Affiliations:** 1 Department of Medicine, Jawaharlal Nehru Medical College, Datta Meghe Institute of Medical Sciences (Deemed to be University), Sawangi (Meghe), Wardha-442001, Maharashtra, India

**Keywords:** Supravalvular aortic stenosis, Williams syndrome, elfin facies

## Image in medicine

Williams syndrome (also known as Williams-Beuren syndrome) is a autosomal dominant disorder associated with deletion of multiple genes on the long arm of chromosome 7. It affects one in every 25000 live births and is associated with elfin facies, cardiac abnormalities like supra valvular aortic stenosis and mental impairment. Williams syndrome is diagnosed in about sixty percent patients of supravalvular aortic stenosis. This patient presented to us with the characteristic ‘elfin facies´ which is shown in the image as having a large mouth, widely spaced eyes, maloccluded teeth, patulous lips broad forehead with a short and upturned nose. Supravalvular aortic stenosis (SVAS) involves a narrowing of the ascending aorta above the level of coronary arteries. Narrowing of the peripheral pulmonary arteries is also present in SVAS with Williams syndrome but it does not progress over time. Clinical findings on examination showed an ejection systolic murmur with no associated ejection click. There was a disparity in the blood pressure between both upper limbs, the right arm systolic blood pressure exceeded that of the left arm by 24 mmHg. The chromosomal defect results in deficiency in elastin. This also results in lax skin and stiff joints with a hoarse voice. Supravalvular AS eventually requires valve replacement when the patient becomes symptomatic.

**Figure 1:**
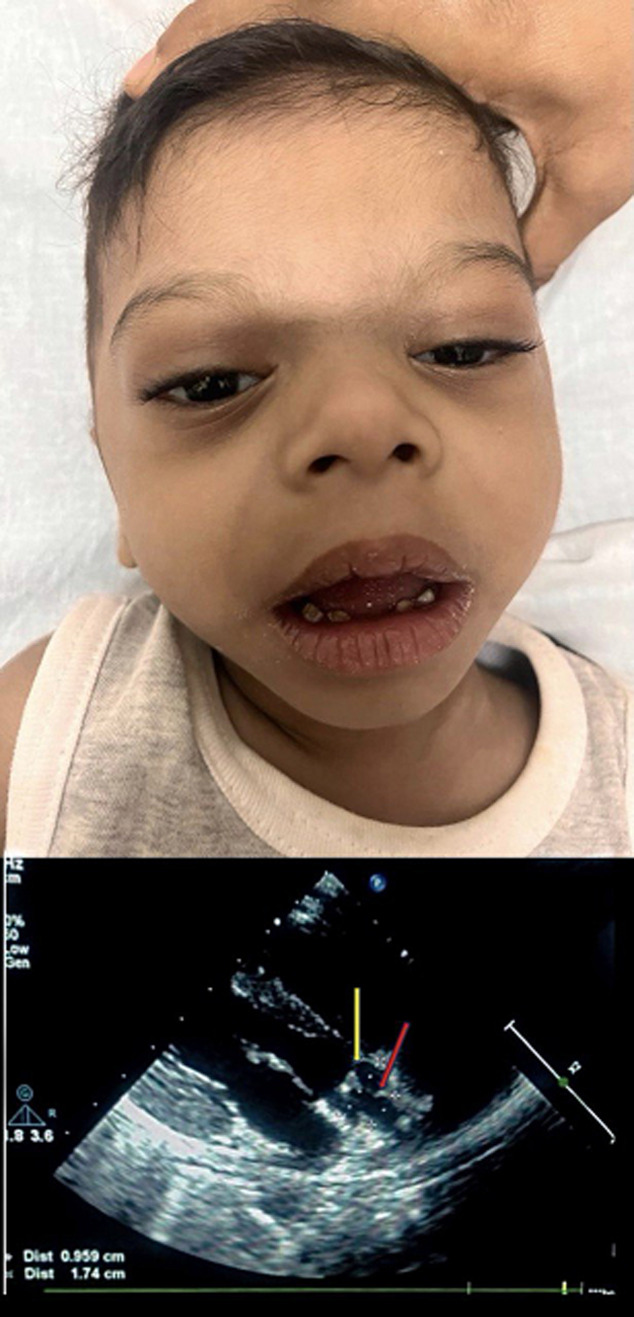
classical elfin facies with 2D echocardiogram (parasternal short axis view) showing supravalvular aortic stenosis (red arrow) and aortic valve (yellow arrow)

